# An Adaptive Leaky-Integrate and Firing Probability Model of an Electrically Stimulated Auditory Nerve Fiber

**DOI:** 10.1177/23312165241286742

**Published:** 2024-11-05

**Authors:** Rebecca C. Felsheim, Mathias Dietz

**Affiliations:** 1 11233 Department of Medical Physics and Acoustics, Carl von Ossietzky Universität Oldenburg, Oldenburg, Germany; 2 597459 Cluster of Excellence “Hearing4All”, Oldenburg, Germany

**Keywords:** auditory model, electrical stimulation, cochlear implant, adaptive threshold neuron model, auditory nerve fiber

## Abstract

Most neural models produce a spiking output and often represent the stochastic nature of the spike generation process via a stochastic output. Nonspiking neural models, on the other hand, predict the probability of a spike occurring in response to a stimulus. We propose a nonspiking model for an electrically stimulated auditory nerve fiber, which not only predicts the total probability of a spike occurring in response to a biphasic pulse but also the distribution of the spike time. Our adaptive leaky-integrate and firing probability (aLIFP) model can account for refractoriness, facilitation, accommodation, and long-term adaptation. All model parameters have been fitted to single cell recordings from electrically stimulated cat auditory nerve fibers. Afterward, the model was validated on recordings from auditory nerve fibers from cats and guinea pigs. The nonspiking nature of the model makes it fast and deterministic while still accounting for the stochastic nature of the spike generation process. Therefore, the relationship between the input to the model or model parameters and the model's output can be observed more directly than with stochastically spiking models.

## Introduction

Computational models deepen our understanding of complex systems, such as the auditory system. Models can even be seen as the most tangible embodiment of our knowledge of the respective system (Frigg, 2006). When modeling a system that includes a stochastic process, such as spike generation in nerve fibers, the model design question arises if the output should be an instance of the process or rather the statistical characterization of the process, as provided by probability distributions.

The more common approach to modeling is to generate an output similar to the observable events. In the case of nerve fibers, it is often the stochastic time points of individual spikes, which results in a spiking model. From the many spiking models of auditory nerve cells, some describe the processes happening in the cell in more detail (e.g., Sumner et al., 2002 and Bruce et al., 2018 for acoustic stimulation; Negm & Bruce, 2014 for electric stimulation), while others focus more on the phenomena that can be observed (e.g., Zilany et al., 2014 for acoustic stimulation; Joshi et al., 2017 for electric stimulation). In this work, we focus on the latter, the phenomenological view of the spike generation process and not the detailed processes in the cell.

Using a nonspiking description of the spike generation in nerve fibers via statistical characterizations of the process, such as spike probability or spike rate over time, is a more abstract approach. However, such models have the advantage of being deterministic and computationally efficient, which allows for the observation of the effect of small changes in the input or in the parameters readily in the output of the model without the potentially obscuring effects of stochasticity while still describing the stochastic properties of the cell.

One important problem in the nonspiking approach occurs in the interaction between spikes. From a phenomenological view, the refractory period starts after a spike is evoked and last for 4 to 10 ms (Dynes, 1996; Miller et al., 2001). During this period, the excitability of the cell is reduced. Additionally, adaptation occurs, which is a long-term reduction of excitability (Zhang et al., 2007), which cannot be explained by refractoriness alone. If a fiber is stimulated without resulting in an action potential, facilitation increases the excitability for up to 0.5 ms (Dynes, 1996), and afterward, the excitability is slightly decreased for up to 5 ms (Dynes, 1996) due to accommodation. The problem with incorporating these phenomena in a nonspiking statistical description of the process is that the response is not a spike, that is, not a binary event, but a firing probability or a spike rate.

Different approaches have been suggested to solve the problem of spike interaction phenomena in nonspiking models. Xu and Collins (2007) looked at the average response of many electrically stimulated auditory nerve fibers and used this to incorporate the refractory period for only a fraction of the simulated nerve fibers, depending on the spike probability. However, rather than evaluating the average spike rate in detail, they computed psychoacoustic measures, such as the dynamic range, from it. With this, they extend the work by Bruce et al. (2000), who estimated the mean response of an electrically stimulated auditory nerve fiber model for stimulation with a uniform pulse train. Verhulst et al. (2018) used a similar approach for a single acoustically stimulated nerve fiber and decreased the spike probability based on the probability at previous time points. In another approach, Augustin et al. (2017) derived differential equations for adaptive integrate and fire models over a period of several hundred milliseconds for a single nerve fiber and time continuous stimuli. Due to this larger time scale, detailed statistics after a single spike are not considered, also because it is difficult to distinguish by which part of the stimulus a spike is exactly evoked.

With electrical stimulation of a nerve cell, as it is done with many neuroprostheses such as cochlear implants, the cells are often stimulated with discrete pulses (Cheng et al., 2017; Wilson et al., 1993), which is a signal that changes fast over time. These discrete pulses provide the unique opportunity to relate each spike to a single causal pulse or to very few pulses. The other way around, we can explore the statistics of the nerve fiber in detail by manipulating the stimulation pulses systematically. Due to the above-mentioned problem with the spike interaction phenomena, many models use spike probability only up to the point where the next input pulse is considered. One example of such a model is the point process model by Goldwyn et al. (2012), where the point process results in an instantaneous spike probability, from which a spike time is then drawn. Using this spike time, the refractory period is implemented for further stimulation. In a similar manner, Pillow et al. (2004) and Monk and Leib (2016) computed the maximum likelihood for a spike time in a general neuron model but needed the exact spike time to consider the refractory period for further stimulation. Campbell et al. (2012) incorporated the pulse interaction phenomena in a nonspiking model of an electrically stimulated auditory nerve fiber by considering both possibilities of a spike occurring and no spike occurring for further computation of the spike probability, similarly as in Verhulst et al. (2018) for acoustically stimulated auditory nerve fibers. However, Campbell et al. (2012) do not compute the detailed statistics of the spike times, only the total spike probability in response to each pulse.

In this work, we extend the idea of Campbell et al. (2012) and propose an adaptive leaky-integrate and firing probability (aLIFP) model. It is a nonspiking, deterministic adaptation of the sequential biphasic leaky-integrate and fire (S-BLIF) model (Takanen & Seeber, 2022b) and provides the instantaneous spike probability over time in response to extracellular stimulation with one or multiple biphasic pulses. The model is parameterized and validated using single cell recordings from cat and guinea pig auditory nerve fibers.

## Model Description

The proposed model is inspired by the S-BLIF model by Takanen and Seeber (2022b) and Horne et al. (2016). The S-BLIF model is a stochastic spiking model that incorporates refractoriness, facilitation, and adaptation in a linear leaky-integrate and fire model, resulting in an adaptive threshold model (Gerstner et al., 2014). We adapted the S-BLIF model into a nonspiking model, by replacing the noisy threshold drawn from a Gaussian distribution with the corresponding distribution. In this way, we can perform all computations directly on the threshold distribution, which allows us to obtain the spike time distribution in response to a biphasic pulse instead of the stochastic spike times. As the aLIFP model also considers the spike interaction phenomena, it falls into the class of adaptive threshold models as well (Gerstner et al., 2014).

**Figure 1.**
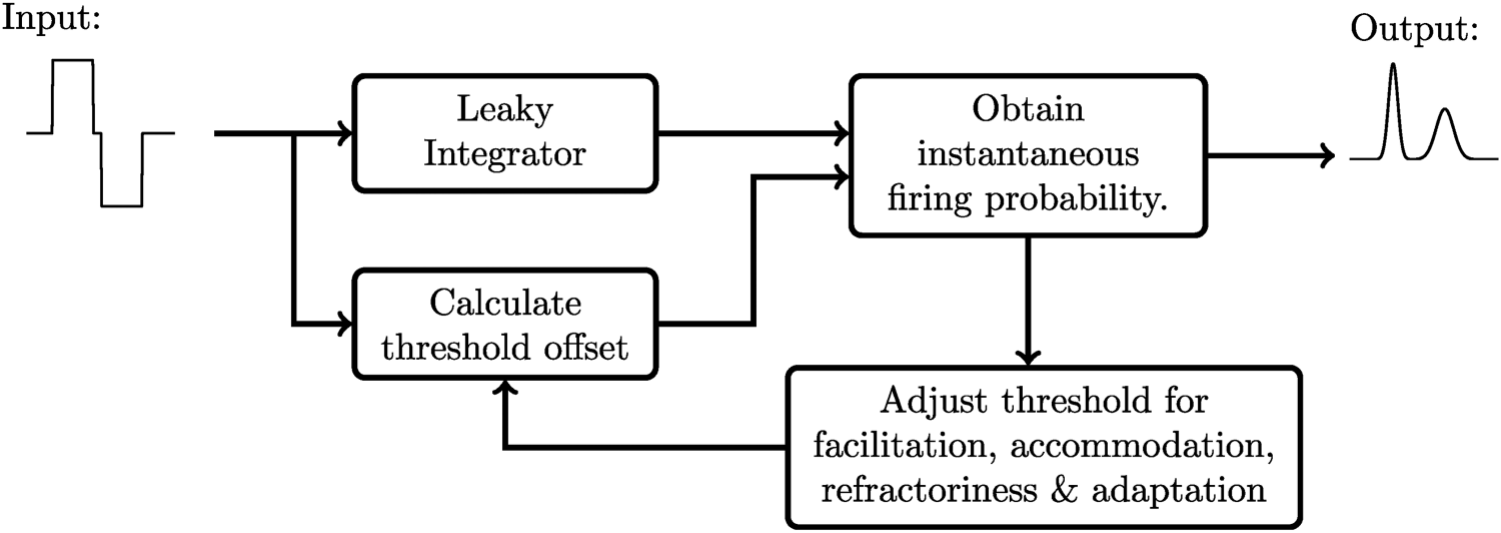
A schematic overview of the aLIFP model. The input to the model are biphasic current pulses. From this, the model obtains its output: the firing probability over time in response to each pulse. aLIFP = adaptive leaky-integrate and firing probability.

Figure 1 gives an overview of our aLIFP model. It consists of four main parts: (a) the leaky integrator; (b) the threshold distribution and the computation of its offset; (c) the calculation of the firing probability and spike time distribution; and (d) the adjustment of the threshold to account for facilitation, accommodation, refractoriness, and adaptation for future stimulation. These parts will be described in more detail in the remainder of this section.

**Figure 2.**
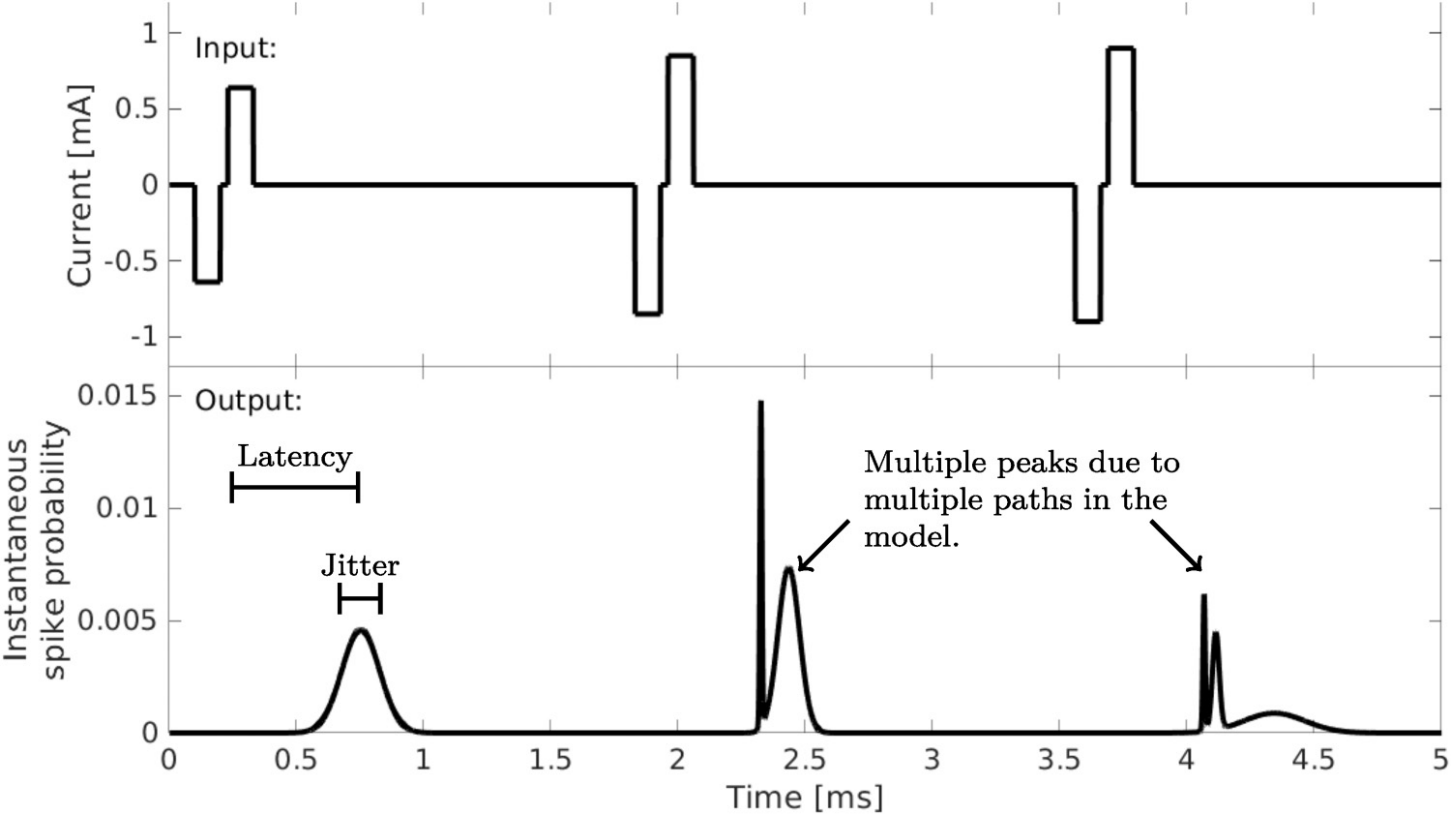
Exemplary input (upper panel) and output (lower panel) of the model.

The input to our model is the extracellular stimulating current. In the present implementation, only biphasic pulses with a rectangular shape can be used as input. The phases do not need to have the same duration or amplitude, and they may be separated by an interphase gap (IPG). Currently, a spike can only be evoked by a cathodic (negative) current, even though in nature spikes can be evoked both by cathodic and anodic stimulation (Shepherd & Javel, 1999). These constraints are not limitations of the nonspiking approach in general, but due to the specific implementation. An exemplary model output can be seen in Figure 2.

### Leaky Integrator

The membrane potential is modeled as a deterministic value that is changed by a stimulating current, as in Horne et al. (2016). The relationship between the stimulating current I(t) and the change in passive membrane potential V(t) is described using a linear leaky integrator (Lapicque, 1907)

(1) $$\tau \frac{d\;V(t)}{d\;t}=-RI(t)-V(t)+V_{Rest}\;.$$

For the sake of simplicity and without loss of generality within the present study, the resting potential *V_Rest_* has been set to 0 V. The membrane resistance *R* was fitted to obtain a physiologically plausible average spike threshold of 10 mV. The electrode-to-neuron interface is not included in the aLIFP model but can be added as a front end. If no electrode-to-neuron interface is added, the membrane resistance can be viewed as a proportionality constant between the current emitted by an electrode and the potential affecting the cell. It can also be chosen as a negative value to describe the stimulation with anodic (positive) extracellular current.

### Threshold and Threshold Offset

In an adaptive leaky-integrate and fire model, a spike is evoked once the membrane potential *V*(*t*) crosses a threshold. This threshold changes over time due to phenomenological spike interaction phenomena such as refractoriness and facilitation, but it is a deterministic value. However, the spike generation process in nature is stochastic, which is not captured by a classical adaptive leaky-integrate and fire model. Therefore, the S-BLIF model describes this stochasticity by drawing the threshold in each time step from a Gaussian distribution (Takanen & Seeber, 2022b). The mean and standard deviation of this Gaussian distribution vary over time to describe the spike interaction phenomena.

Instead of drawing samples from a distribution, in the aLIFP model, all calculations are directly done on the Gaussian distribution describing the threshold, given as

(2) $$\theta (t)=N(\mu_{\theta }(t),\sigma_{\theta }(t))\;.$$

The Gaussian distribution N is described by a time varying mean $\mu_{\theta }(t)$ and a time varying standard deviation $\sigma_{\theta }(t)$ . The default values for the mean and standard deviation are $m_{\theta }$ and $s_{\theta }$ , respectively.

To evoke a spike with the same probability, a higher amplitude is needed for a biphasic pulse compared to a monophasic one (Shepherd & Javel, 1999). In exponential leaky-integrate and fire models, this mechanism is inherently included (Brette & Gerstner, 2005). However, in models based on a linear leaky integrator, a separate mechanism is needed. Here, it can be seen as the second phase preventing the generation of an action potential, even though the charge from the first phase would be sufficient to cause a spike (Horne et al., 2016). The period during which the spike prevention can happen has been dubbed by Horne et al. (2016) the “action potential initiation phase” with the duration φ , which starts after the membrane potential has crossed the threshold. To cancel the spike generation in the S-BLIF model, the total current delivered during the action potential initiation phase must become negative. As our threshold is not a deterministic value, we transform this spike generation cancellation into an equivalent increase in the threshold, dependent on the pulse shape. In the following, it will be explained how such a threshold offset can be obtained if the pulse has two rectangular phases. This transformation of the estimation of the spike cancelation mechanism proposed by Horne et al. (2016) into a mechanism that can be used with our nonspiking model is the reason why we had to limit the input of our model to biphasic pulses with rectangular phases.

The duration of the action potential initiation phase φ can be split into a part $\varphi_{p}$ during the positive phase, $\varphi_{n}$ during the negative one, and the duration of the IPG $d_{\mathrm{IPG}}$ :

(3) $$\varphi =\varphi_{p}+\varphi_{n}+d_{\mathrm{IPG}}\;.$$

With the amplitudes $a_{p}$ and $a_{n}$ of the positive and negative phase of a biphasic pulse and the assumptions made by Takanen and Seeber (2022b), spike cancellation happens if the charge $a_{p}\cdot \varphi_{p}$ delivered during the positive part of the action potential initiation period is larger than the charge $a_{n}\cdot \varphi_{n}$ delivered during the negative one. A prerequisite for spike cancellation is, therefore,

(4) $$a_{p}\cdot \varphi_{p}>a_{n}\cdot \varphi_{n}\;.$$

For biphasic pulses with equal amplitudes $a_{n}=a_{p}$ , equation (4) simplifies to

(5) $$\varphi_{p}>\varphi_{n}.$$

Equations (3) and (4) can be combined to get the minimally necessary duration of the action potential initiation phase during the negative pulse phase $\varphi_{n,\min}$ to successfully evoke a spike:

(6) $$\varphi_{n,\min}=\frac{\varphi -d_{IPG}}{1+\frac{a_{n}}{a_{p}}}\;.$$

Again, equation (6) can be simplified to:

(7) $$\varphi_{n,\min}=\frac{\varphi -d_{IPG}}{2}$$

if both phases have the same amplitude.

To prevent the cancellation of the action potential generation, the threshold must be reached $\varphi_{n,\min}$ before the end of the negative phase. This is given as:

(8) $$t_{\mathrm{cancel}}=t_{\mathrm{start}}+d_{p}-\varphi_{n,\min}\;,$$

where $d_{p}$ is the total duration of the positive phase and $t_{\mathrm{start}}$ the starting point of the pulse.

Instead of enforcing a threshold crossing before $t_{\mathrm{cancel}}$ directly, an equivalent threshold offset that needs to be reached until the end of the cathodic phase is defined. This simplification is possible, as with rectangular pulses, the modeled membrane potential is strictly increasing until the end of the cathodic phase ( $t_{\mathrm{start}}+d_{n}$ ). The threshold offset $o_{\mathrm{cancel}}$ is defined as the distance between the maximum value of the membrane potential $V_{\max}$ and the membrane potential at the time point $t_{\mathrm{cancel}}$ . It can thus be obtained as:

(9) $$o_{\mathrm{cancel}}=V_{\max}-V(t_{\mathrm{cancel}})\;.$$

**Figure 3.**
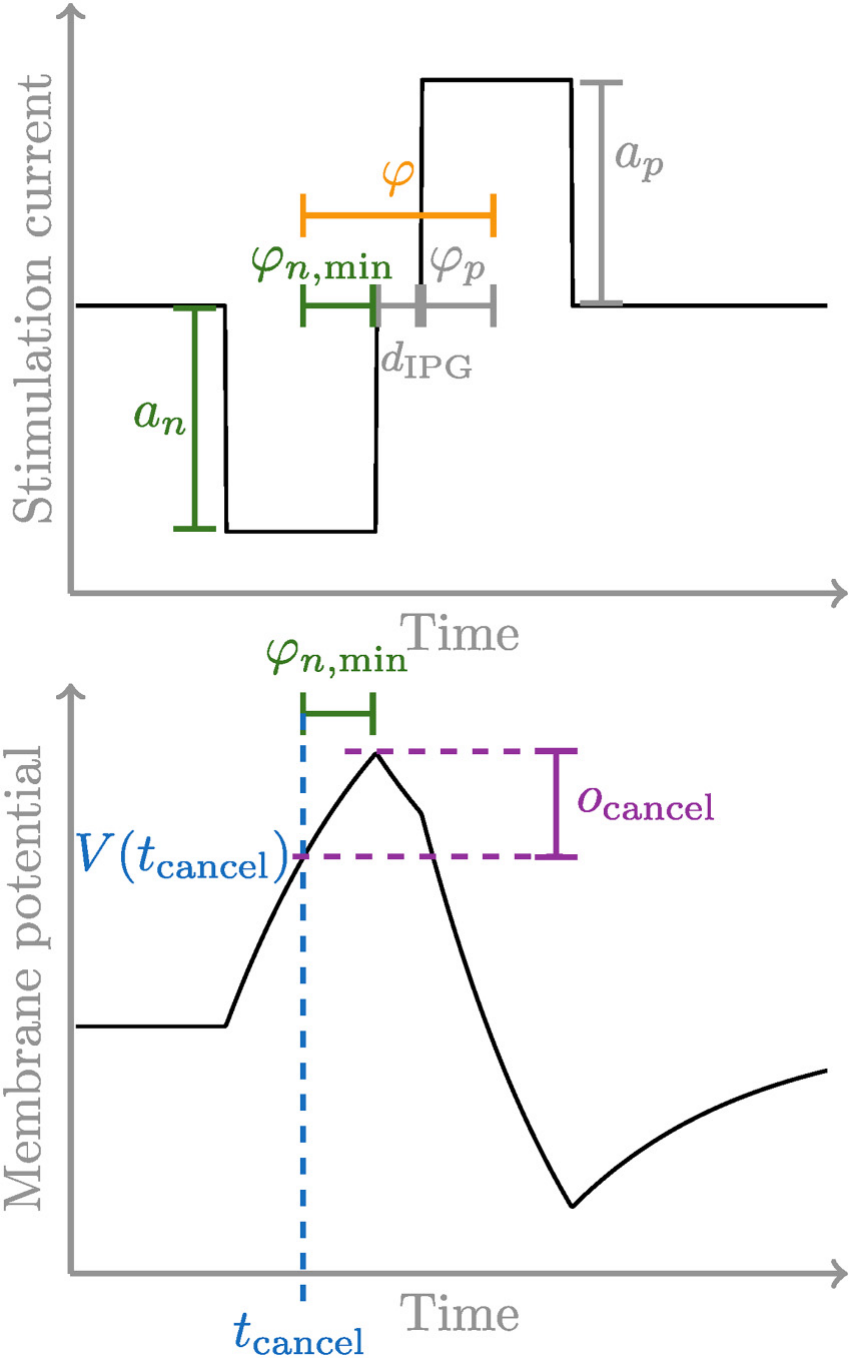
A visualization of how the threshold offset is obtained. First $\varphi_{n,\min}$ is calculated using equation (4) and from this $V(t_{\mathrm{cancel}})$ and the offset $o_{\mathrm{cancel}}$ are obtained.

An illustration of the calculation of the threshold offset can be found in Figure 3.

Finally, the effective threshold can be obtained as

(10) $$\theta_{\mathrm{eff}}(t)=\theta (t)+o_{\mathrm{cancel}}=N(\mu_{\theta }(t)+o_{\mathrm{cancel}},\sigma_{\theta }(t))\;.$$

### Firing Probability

The firing probability is the probability of the membrane potential being higher than the threshold distribution. For a constant threshold, this probability is modeled as (Horne et al., 2016)

(11) $$P_{s}=\Phi \left(\frac{V_{\max}-\mu_{\theta }}{\sigma_{\theta }}\right)\;,$$

where Φ(⋅) denotes the standard cumulative Gaussian distribution, which is the integral over the Gaussian distribution. However, the threshold is not constant, and the maximal distance between the membrane potential and the threshold, and thus highest spike probability, is not necessarily at the local maximum of the potential. Therefore, the maximum firing probability during the stimulation with a pulse is used:

(12) $$P_{s}=\max\;\mathrm{Pr}\{V(t)\geq \theta (t)\}\;.$$

For this, the firing probability at each time point is obtained, and then the maximum over time is used. This results in

(13) $$P_{s}=\max\;\Phi \left(\frac{V(t)-\mu_{\theta }(t)}{\sigma_{\theta }(t)}\right)\;\mathrm{for}\;t\in [t_{\mathrm{start}},t_{\mathrm{stop}}]\;,$$

where $t_{\mathrm{start}}$ is the start time of the pulse and $t_{\mathrm{stop}}$ the end time of the first pulse phase. Incorporating the increase in the threshold due to spike cancellation changes the spike probability to

(14) $$\begin{array}{rl} \;P_{s}= & \max\mathrm{Pr}\{V(t)\geq (\theta (t)+o_{\mathrm{cancel}})\}\\ \;= & \max\Phi \left(\frac{V(t)-(\mu_{\theta }(t)+o_{\mathrm{cancel}})}{\sigma_{\theta }(t)}\right)\;\mathrm{for}\;t\in [t_{\mathrm{start}},t_{\mathrm{stop}}]\;, \end{array}$$

where $o_{\mathrm{cancel}}$ is the voltage offset as described in equation (9).

### Spike Time Distribution

The second part of the aLFIP model output is the distribution of the spike time in response to each pulse. Like the threshold, the spike time (st) distribution is modeled as a Gaussian distribution:

(15) $$D_{\mathrm{st}}=N(\mu_{\mathrm{st}},\sigma_{\mathrm{st}})\;.$$

The Gaussian distribution is chosen here for simplicity, even though the distribution of measured spike times is not necessarily a Gaussian distribution (Javel & Shepherd, 2000). However, this does not limit our approach, as will be discussed later. The distribution is obtained by combining the threshold crossing time (ct) distribution

(16) $$D_{ct}=N(\mu_{ct},\sigma_{ct})$$

with the latency. The latency is also modeled as a Gaussian distribution with the mean $\mu_{L}$ and standard deviation $\sigma_{L}$ , also called jitter. As both the latency and the jitter change with the stimulating amplitude (Miller et al., 1999), the mean of the latency is described as (Takanen & Seeber, 2022b)

(17) $$\begin{array}{r} \mu_{L}=l_{3}\cdot {\left[1+\exp\;\left(\frac{(V_{\max}-(\mu_{\theta }(\mu_{ct})+o_{\mathrm{cancel}}))-l_{1}}{l_{2}}\right)\right]}^{-1}+l_{4}, \end{array}\;$$

and the jitter as (Takanen & Seeber, 2022b)

(18) $$\begin{array}{r} \sigma_{L}=j_{3}\cdot {\left[1+\exp\;\left(\frac{(V_{\max}-(\mu_{\theta }(\mu_{ct})+o_{\mathrm{cancel}}))-j_{1}}{\;j_{2}}\right)\right]}^{-1} \end{array}.$$

The latency is parameterized with $l_{1},l_{2},l_{3}$ , and $l_{4}$ and the jitter with $j_{1},j_{2}$ , and $j_{3}$ . In contrast to Takanen and Seeber (2022b), the latency and jitter are dependent on the maximum membrane potential $V_{\max}$ instead of the spike probability. This allows both values to decrease further even if a spike probability of 1 is reached, as indicated by data from Sly et al. (2007).

The threshold crossing distribution is obtained using the Gaussian-like function

(19) $$g(t)=\Phi \left(\frac{V(t)-\mu_{\theta }(t)}{\sigma_{\theta }(t)}\right)\;\mathrm{for}\;t\in [t_{\mathrm{start}},t_{\mathrm{stop}}]\;,$$

which is similar to the one describing the spike probability, except that spike cancellation is not considered here. If the membrane potential V(t) would be linearly rising, g(t) would be a cumulative Gaussian distribution. However, as the membrane potential is in not exactly linearly rising, the mean $\mu_{ct}$ and the standard deviation $\sigma_{ct}$ are found as the parameters of a Gaussian cumulative distribution fit in g(t) .

Finally, the mean and standard deviation of the spike time distribution $D_{\mathrm{st}}$ are obtained by adding the two Gaussian variables described by the latency distribution and threshold crossing distribution, resulting in

(20) $$\begin{array}{rl} \mu_{st}= & \mu_{ct}+\mu_{L}\\ \sigma_{st}= & \sqrt{\sigma_{ct}^{2}+\sigma_{L}^{2}}\;. \end{array}$$

### Change in Threshold After a Pulse

The spike interaction phenomena are incorporated similarly as in Campbell et al. (2012). Refractoriness and adaptation occur with the probability of firing $P_{s}$ , and facilitation and accommodation with the probability of not firing $1-P_{s}$ . To describe this, the threshold is split into two paths: one describing threshold change due to refractoriness and adaptation, weighted with the probability of firing $P_{s}$ , and the other one describing the change due to facilitation and accommodation, weighted with the probability of not firing $1-P_{s}$ . After every pulse, the number of paths is duplicated in this way. All calculations described above are done separately for each path, and therefore, one firing probability $P_{s,k}$ for each path is obtained. The total firing probability is then found by adding the spike probabilities of all *K* paths:

(21) $$P_{s}=\overset{K}{\underset{k=1}{\sum }}\;P_{s,k}\;.$$

For each path, its probability of occurring, or weight, $w_{k}$ is obtained. Before the first pulse is presented, only one path exists, and its weight is 1. After the first pulse, we have two paths with the weights

(22) $$\begin{array}{rl} & w_{1}=P_{s}\\ & w_{2}=1-P_{s} \end{array}.$$

To obtain the weights after the second pulse, the two existing paths need to be considered. Each existing path is split into two new paths: one accounting for refractoriness and adaptation and one accounting for facilitation and accommodation, which results in four paths. In general, the weights of each new pair of paths can be obtained by multiplying the weight of the current path ${\hat{w}}_{k}$ with the probability of firing or not firing:

(23) $$\begin{array}{rl} & \;\;\;\;\;\;w_{k}={\hat{w}}_{k}\cdot P_{s,k}\\ & w_{\hat{K}+k}={\hat{w}}_{k}\cdot (1-P_{s,k}) \end{array}\;,$$

where $\hat{K}$ is the old number of threshold paths. In this way, the number of paths is doubled after each pulse. As the weights describe the probability with which each path occurs, all weights must sum up to 1.

Mathematically, the multiple paths of the threshold can be described with a Gaussian mixture distribution. For this, the Gaussian distribution describing each threshold path is multiplied with the corresponding weight, or probability of occurring, and these weighted Gaussian distributions are summed up. The total distribution of the threshold can then be written as:

(24) $$\begin{array}{r} \theta (t)=\overset{K}{\underset{k=1}{\sum }}\;w_{k}\cdot N(\mu_{\theta ,k}(t),\sigma_{\theta ,k}(t)),\\ \mathrm{with}\;\overset{K}{\underset{k=1}{\sum }}\;w_{k}=1,w_{k}\geq 0\;. \end{array}$$

Each Gaussian distribution has its own mean $\mu_{\theta ,k}(t)$ and standard deviation $\sigma_{\theta ,k}(t)$ , dependent on the path taken through the spike and no spike possibilities.

As a crossing time distribution $D_{ct}$ and a spike time distribution $D_{\mathrm{st}}$ for each path is obtained, the final spike time distribution is also a weighted sum of Gaussian distributions, or a Gaussian mixture, with the same number of components as the threshold prior to stimulation. For each component, the mean and standard deviation of the spike time distribution are obtained as described before. Each of them has again a weight, or probability of occurring, $w_{\mathrm{st},k}$ . They can be found by multiplying the weights of the threshold with the spike probability $P_{s,k}$ in the corresponding path and rescaling them such that they sum up to 1.

To avoid an exponentially increasing number of components, the implementation enforces a maximum number of components $K_{\max}$ . For this, two rules are applied. First, if the mean and standard deviation of two components are the same, they are combined into a single one. Second, if θ(t) contains more than $K_{\max}$ components, the components with the lowest weights are removed until only $K_{\max}$ components are left. Then, all other weights are rescaled.

In the following, it will be explained how the mean and standard deviation are changed, leading $\mu_{\mathrm{fac},\mathrm{acc}}(t)$ and $\sigma_{\mathrm{fac},\mathrm{acc}}(t)$ due to facilitation and accommodation and to $\mu_{\mathrm{ref},\mathrm{ada}}(t)$ and $\sigma_{\mathrm{ref},\mathrm{ada}}(t)$ due to refractoriness and adaptation.

### Facilitation and Accommodation

**Figure 4.**
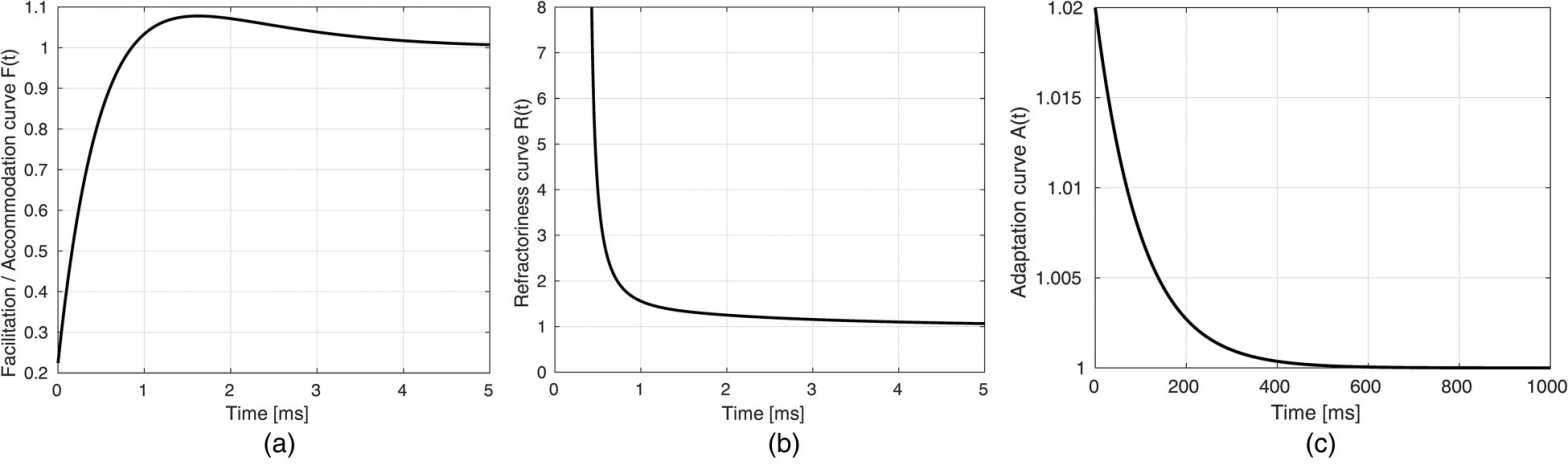
Exemplary plots of the functions used to describe (a) facilitation and accommodation, (b) refractoriness, and (c) adaptation. The exact function course depends on the chosen parameter values.

In Takanen and Seeber (2022b), only facilitation is modeled. For biphasic pulses, however, accommodation-like behavior is caused by the leaky integrator model, as the second phase hyperpolarizes the membrane, some of which remains until the start of the next pulse. It is not actual accommodation of the neural response properties. Here, we include both facilitation and accommodation as time-dependent neural properties using a single function. This function is designed to be multiplied with the threshold, similar as in Takanen and Seeber (2022b). Therefore, a function is needed that is first smaller than 1 but larger than 0 to decrease the threshold, then increases above 1 for a certain amount of time, and finally converges to 1. The resulting function has been inspired by data from Dynes (1996) and can be given as

(25) $$F(t)=(1-e^{-f_{4}\cdot (t+f_{1})})\cdot (1+f_{3}\cdot e^{-f_{4}\cdot (t+f_{2})})\;,$$

where $f_{1},f_{2},f_{3}$ , and $f_{4}$ are fit parameters. A graph of F(t) can be seen in Figure 4a, and it shows that it fulfills the requirements stated above. The function describing facilitation and accommodation is applied to the mean of the threshold from the end point $t_{\mathrm{stop}}$ of the first phase onward using

(26) $$\mu_{\mathrm{fac},\mathrm{acc}}(t)=\mu_{\theta }(t)\cdot F(t-t_{\mathrm{stop}})\;.$$

As facilitation can accumulate for multiple subthreshold stimulation, a lower bound of $f_{5}\cdot m_{\theta }$ is placed on $\mu_{\mathrm{fac},\mathrm{acc}}(t)\;$ to avoid implausibly low values of the threshold, which could result in a spike probability larger than 0 without stimulation. Applying F(t) in this way allows for an accumulation of facilitation or accommodation as found by Dynes (1996). The standard deviation is obtained similarly, resulting in

(27) $$\sigma_{\mathrm{fac},\mathrm{acc}}(t)=\sigma_{\theta }(t)\cdot F(t-t_{\mathrm{stop}})\;.$$

A lower bound of $f_{5}\cdot s_{\theta }$ is also placed on the standard deviation.

### Refractory Period

The refractory curve is modeled with the same function as in Takanen and Seeber (2022b), which was first published by Hamacher (2003):

(28) $$R(t)=\{\begin{array}{c} \infty \\ {\left[\left(1-\exp\;\left(\frac{-t+t_{\mathrm{ARP}}}{q\tau_{\mathrm{RRP}}}\right)\right)\left(1-p\exp\;\left(\frac{-t+t_{\mathrm{ARP}}}{\tau_{\mathrm{RRP}}}\right)\right)\right]}^{-1} \end{array}\;\;\;\mathrm{if}\;\;\;\;\;\;\;\;\begin{array}{c} t<t_{\mathrm{ARP}}\\ t\geq t_{\mathrm{ARP}} \end{array}\;,$$

where $t_{\mathrm{ARP}}$ is the absolute refractory period, $\tau_{\mathrm{RRP}}$ the time constant of the relative refractory period, and *p* and *q* are two parameters to describe the shape of the refractory curve. A graph of the function course can be seen in Figure 4b. It shows that R(t) has very high values at the beginning and then converges to 1. It is applied to the threshold after the mean time point of threshold crossing $\mu_{ct}$ , similarly as in Takanen and Seeber (2022b):

(29) $$\mu_{ref}(t)=\mu_{\theta }(t)\cdot R(t-\mu_{ct})\;.$$

The change in the standard deviation is obtained in the same way as the change in the mean of the threshold:

(30) $$\sigma_{ref}(t)=\sigma_{\theta }(t)\cdot R(t-\mu_{ct})\;.$$

To avoid a large spike probability for an amplitude of 0, we set a lower limit to the standard deviation of $\mu_{\theta }(t)\cdot b$ . Currently, *b* is set to 1/3 , which avoids a spike probability larger than 0.1% for an amplitude of 0. This value was chosen as it is commonly used in statistics as a border for very unlikely events (Janczyk & Pfister, 2023).

### Adaptation

Adaptation is modeled by multiplying a function A(t) , which describes the increase in the threshold, with the mean threshold. The adaptation function (Takanen & Seeber, 2022b) is given as

(31) $$A(t)=1+c_{a}\cdot \exp\;\left(-\frac{t}{\tau_{a}}\right)\;.$$

The time constant $\tau_{a}$ describes the duration of the exponential decay, and $c_{a}$ the maximum increase due to each individual pulse. A graph of the curve can be seen in Figure 4c. It shows that the increase due to adaptation is very small but much longer compared to the changes due to refractoriness, facilitation, and accommodation.

Finally, the adaptation function is applied onto the mean threshold value after the time point of threshold crossing $\mu_{ct}$ , which results in

(32) $$\mu_{ref,ada}(t)=\mu_{ref}(t)\cdot A(t-\mu_{ct})\;.$$

The total increase due to adaptation is limited by $a_{\max}$ . Similarly, the standard deviation of the threshold is changed to

(33) $$\sigma_{ref,ada}(t)=\sigma_{ref}(t)\cdot A(t-\mu_{ct})\;.$$

## Model Fitting

The aim of our work was to provide an alternative model output representation, namely spike time distributions. It was not the aim to improve the prediction of data or to make a more realistic model of specific processing stages in general. To ensure the applicability of the aLIFP model, all parameters were found by fitting single cell recordings of cat auditory nerve fibers from the literature. Independent of the pulse polarity in the studies, we used pulses with a negative leading phase, as only those can evoke a spike in our model. The final parameter values can be found in Table 1. All predictions shown were made with this set of parameters. For comparison, many figures also show the predictions by the S-BLIF model (Takanen & Seeber, 2022b), as it was our aim to create a nonspiking model that performs similarly to the S-BLIF model for biphasic pulses with a negative leading phase. The data for fitting and validation were read out by us from the figures of the original publications, except for the data from Cartee et al. (2000), Dynes and Delgutte (1992), refractory data from Dynes (1996), Hartmann and Klinke (1990), Heffer et al. (2010), Javel (1990), Miller et al. (2008), and Miller et al. (2011), which were published digitally, together with the S-BLIF model on Zenodo (Takanen & Seeber, 2022a).

**Table 1.** All Parameters of the aLIFP Model, Together With Their Value Found During Fitting and Used During the Validation.

Symbol	Description	Value
τ	Membrane time constant	120 µs
R	Membrane resistance	28*.*99 Ω
$m_{\theta }$	Starting value of the threshold mean (chosen)	10 mV
$s_{\theta }$	Starting value of the threshold standard deviation	0*.*43 mV
φ	Duration of the action potential initiation period	20*.*5 µs
$l_{1}$	Latency parameters	110 µV
$l_{2}$		548 µV
$l_{3}$		393 µ
$l_{4}$		423 µs
$j_{1}$	Jitter parameters	545 µV
$j_{2}$		316 µV
$j_{3}$		130 µs
$f_{1}$	Facilitation and accommodation parameters	0.1 ms
$f_{2}$		−1.4 ms
$f_{3}$		0.45
$f_{4}$		0.9 ms^−1^
$f_{5}$		0.5
$t_{\mathrm{ARP}}$	Duration of the absolute refractory period	0*.*37 ms
$\tau_{\mathrm{RRP}}$	Duration of the relative refractory period	2*.*56 ms
q	Refractoriness parameter	0.102
p	Refractoriness parameter	0.377
$a_{\max}$	Maximum adaptation	1.7
$c_{a}$	Adaptation increase	0.015
$\tau_{a}$	Adaptation time constant	0.27 s

**Figure 5.**
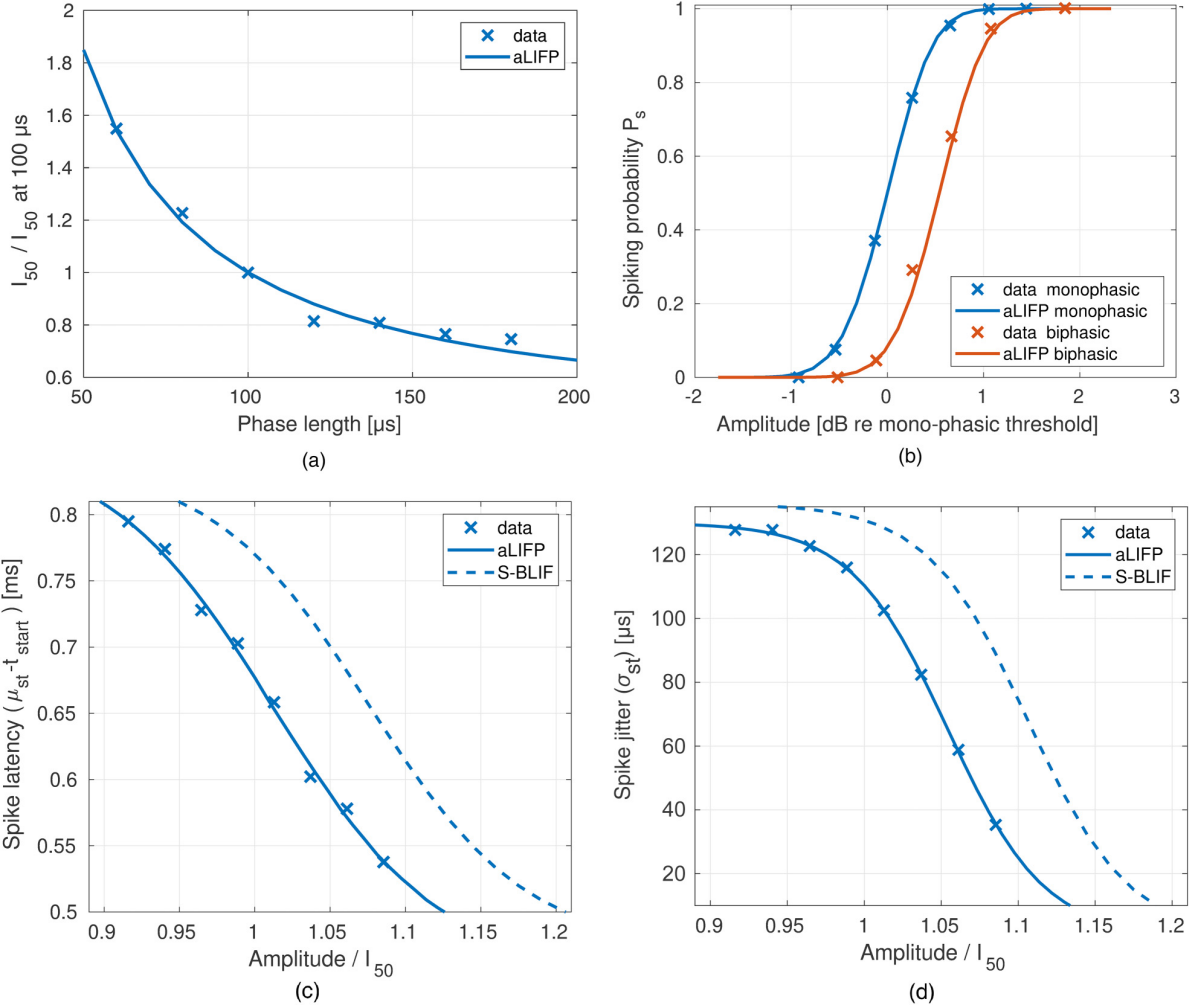
The upper two panels show data by Shepherd and Javel (1999) together with the prediction of the aLIFP model. (a) The $I_{50}$ (current amplitude at 50% firing probability) of a single cat nerve fiber for biphasic pulses with different phase durations are shown. It is normalized on the *I*_50_ of the pulse with 100 µs phase duration. (b) The spike probability of a second nerve fiber for mono- and biphasic pulses with phase durations of 100 µs and an interphase gap of 0 µs is shown. In the lower two panels, data describing the spike latency (c) and spike jitter (d) are shown. These data were collected by Miller et al. (1999), who used monophasic pulses with a duration of 40 µs and without an interphase gap. aLIFP = adaptive leaky-integrate and firing probability; S-BLIF = sequential biphasic leaky-integrate and fire.

For fitting the basic input–output parameters of the model, data by Shepherd and Javel (1999) were used. They recorded the rate-level functions of cat nerve fibers for various pulse shapes. First, the membrane time constant τ from equation (1) was fitted on the spike probability of a single nerve fiber for biphasic pulses with different phase durations and without an IPG. For this, we define $I_{50}$ as the pulse amplitude where a spike is evoked with a probability of 50% ( $P_{s}=0.5$ ). The time constant was fitted to the data normalized to the $I_{50}$ for a pulse with 100 µs phase duration. The results are shown in Figure 5a. As the S-BLIF model was fitted on a completely different data set, it is not included in the figure.

Afterward, the firing probability over amplitude was fitted on monophasic pulses with a phase duration of 100 µs of another nerve fiber from the same publication (Shepherd & Javel, 1999). As the mean value of the threshold $m_{\theta }$ was chosen as 10 mV, the membrane resistance *R*, as a scaling constant, and the standard deviation $s_{\theta }$ were fitted on it. Finally, the duration of the action potential initiation period φ , or maximum time of spike cancellation, has been fitted on the rate-level function with biphasic stimulation of the same nerve fiber. The results of this fit can be seen in Figure 5b.

We fitted the parameters describing the mean latency ( $l_{1},l_{2},l_{3}$ , and $l_{4}$ ; equation (17)) and its standard deviation ( $j_{1},j_{2}$ , and $j_{3}$ ; equation (18)) on data collected by Miller et al. (1999). They stimulated auditory nerve fibers in cats with monophasic pulses with a duration of 40 µs and measured the responses. In their data, the distance between the mean spike time and the starting point of the pulse $\mu_{\mathrm{st}}-t_{\mathrm{start}}$ was measured as spike latency and the standard deviation $\sigma_{st}$ of the spike time as spike jitter. Figure 5c and d show that the predictions of the aLIFP model fit the data nicely and that there is a slight shift between the S-BLIF model predictions and the data. This shift can be explained by the fact that the latency and jitter of the S-BLIF model were fitted over absolute, not relative, amplitude and that the rate-level function of the S-BLIF model, relating the absolute to the relative amplitude, was fitted on different data.

The parameters describing the refractory curve (equation (28); $p,q,t_{\mathrm{ARP}}$ , and $\tau_{\mathrm{RRP}}$ ) were fitted on data from Dynes (1996) and Miller et al. (2001). Dynes (1996) used 100 µs monophasic pulses to estimate the change in $I_{50}$ in eight cat nerve fibers during the refractory period, and Miller et al. (2001) used 40 µs monophasic pulses to estimate the change in $I_{50}$ during refractoriness in 34 cat nerve fibers. Both data sets were used, as Miller et al. (2001) measured more data points at the beginning of the refractory period and Dynes (1996) measured up to 12 ms. Additionally, Miller et al. (2001) estimated the standard deviation of a cumulative Gaussian function describing the rate-level function during the refractory period. This standard deviation divided by the $I_{50}$ is called the relative spread (Miller et al., 1999).

**Figure 6.**
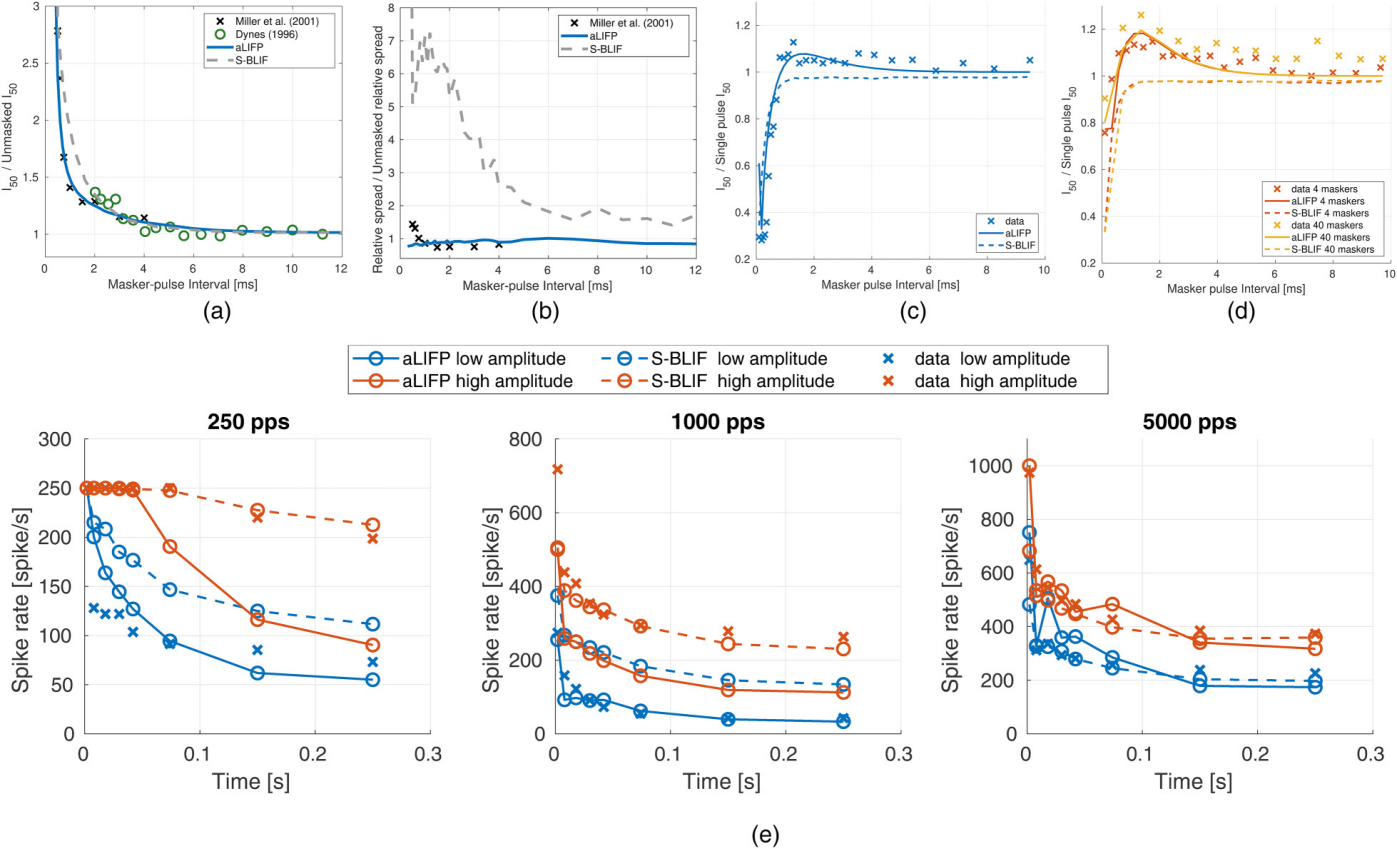
The mean $I_{50}$ (a) and relative spread (b) during the refractory period recorded with monophasic pulses with a length of 40 µs in 34 cat nerve fibers (Miller et al., 2001) and a length of 100 µs in eight cat nerve fibers by Dynes (1996), together with the predictions made by the aLIFP and S-BLIF model. (c) The data by Dynes (1996) for facilitation by a single subthreshold pulse are shown, again, together with the predictions by both models, (d) the same is shown for four and 40 subthreshold maskers. Dynes (1996) used monophasic pulses with a duration of 100 µs. (e) The spike rate adaptation for different pulse rates and amplitudes, as measured by Zhang et al. (2007). Our model was fitted at two amplitudes for each of the pulse rates 250 pps, 1,000 pps, and 5,000 pps. The low amplitude was always 1.4 dB re $I_{50}$ . For 250 pps and 1,000 pps the high amplitude was 3.1 dB re $I_{50}$ and for 5,000 pps the high amplitude was 4.0 dB re $I_{50}$ . Note the slightly higher second amplitude for 5,000 pps, which was used according to the data. Together with the predictions from the aLIFP model, the plots also show the predictions from the S-BLIF model. aLIFP = adaptive leaky-integrate and firing probability; S-BLIF = sequential biphasic leaky-integrate and fire.

The mean $I_{50}$ and relative spread data, together with our predictions and the ones from the S-BLIF model, can be seen in Figure 6a and b. It shows the aLIFP model describes the decay in $I_{50}$ accurately, and there is a small deviation between the data and the predictions by the S-BLIF model (Takanen & Seeber, 2022b). This has been addressed in a personal communication with the authors and is due to a mistake in extraction of the original refractoriness data from Miller et al. (2001) for the basis of fitting the model parameters for refractoriness.

The small changes in relative spread cannot be described by our model; instead, the relative spread stays constant. To describe these small changes, further model parameters would be needed. The S-BLIF model predicts a higher relative spread during the refractory period, which does not match the data. However, the changes in relative spread were not part of the S-BLIF model design.

The data from Dynes (1996) were used to fit the parameters $f_{1},f_{2},f_{3},f_{4}$ , and $f_{5}$ of equation (25). These data were collected using monophasic pulses with 100 µs phase duration. The predictions of both models together with the data can be seen in Figure 6c. The S-BLIF model can predict the decrease in $I_{50}$ up to 0.5 ms due to facilitation nicely. As accommodation was not included in the model, the increase in $I_{50}$ cannot be described.

Additionally, Dynes (1996) also measured the threshold after four and 40 subthreshold pulses. Our model was only adapted to this by allowing an accumulation of facilitation and accommodation. No additional parameters were fitted. Nevertheless, the aLIFP model can reproduce the increase of the $I_{50}$ after four maskers, spaced 1 ms apart. However, it underestimates the effect for 40 maskers. The predictions to both can be seen in Figure 6d.

As a last step during fitting, the maximum adaptation $a_{\max}$ , the adaptation increase $c_{a}$ , and the time constant of the adaptation $t_{a}$ of equation (32) were fitted to the data by Zhang et al. (2007). They measured the spike rate of cat auditory nerve fibers in response to pulse trains consisting of biphasic pulses with 40 µs phase duration and no IPG. The data used for fitting consist of the responses of a single nerve fiber to three different pulse rates, 250 pps, 1,000 pps and 5,000 pps, at two different levels. For 5,000 pps, the second level was slightly higher compared to 250 pps and 1,000 pps. The spike rate of the aLIFP model was obtained by summing the spike probabilities during the time window of interest. Only the absolute amplitude but not the stimulus levels relative to the $I_{50}$ of the cat nerve fiber were known. However, as the predictions of the aLIFP model are sensitive to small changes in the amplitude and the absolute stimulus amplitudes were not compatible with our previously fitted parameters, we fit the amplitudes together with the model parameters while keeping their relative distance in dB constant.

The predictions of the aLIFP model are shown in Figure 6e together with the predictions of the S-BLIF model. Both models perform similarly well, with the aLIFP model being closer to the data for some conditions, while for others, the S-BLIF model performs better. These differences can be explained by the dependence of the predictions on all parameters of the model, which leads to difficulties in fitting these data.

## Model Validation

After the model was fitted, it was validated on additional data sets to test the generalizability of the parameters within the limits of biphasic pulses and a negative leading phase. Additionally, the performance was compared with the S-BLIF model and to ensure that both models perform similarly. As before, all simulations were conducted using the same model parameters.

**Figure 7.**
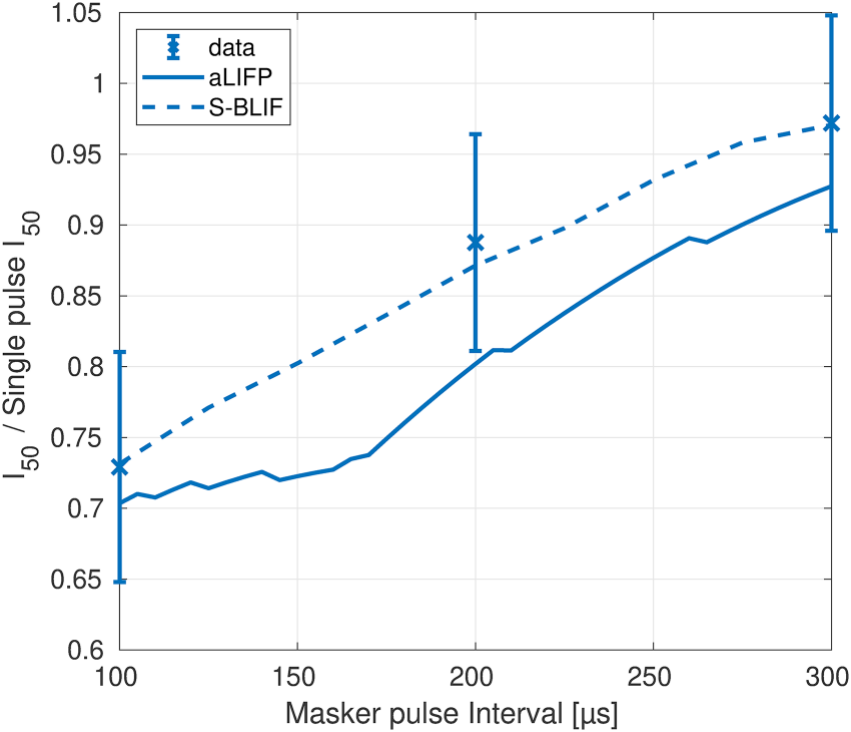
The decrease in threshold due to facilitation is measured by Cartee et al. (2000) by dividing the $I_{50}$ of two pseudo-monophasic pulses by the $I_{50}$ of a single of these pulses. The data were collected by Cartee et al. (2000) using 38 cat nerve fibers and are compared to predictions of the aLIFP model and the S-BLIF model. aLIFP = adaptive leaky-integrate and firing probability; S-BLIF = sequential biphasic leaky-integrate and fire.

The first validation addresses the facilitation, as measured by Cartee et al. (2000). They used pseudo-monophasic pulses to stimulate 38 cat nerve fibers. Both pulses always had the same amplitude, and they measured the reduction in the threshold of the two pulses compared to a single pulse with the same shape. Figure 7 shows that the S-BLIF model fits the data nicely, which was expected as it was fit on this data, while the aLIFP model predicts the data less well, but it is still able to simulate the general trend.

**Figure 8.**
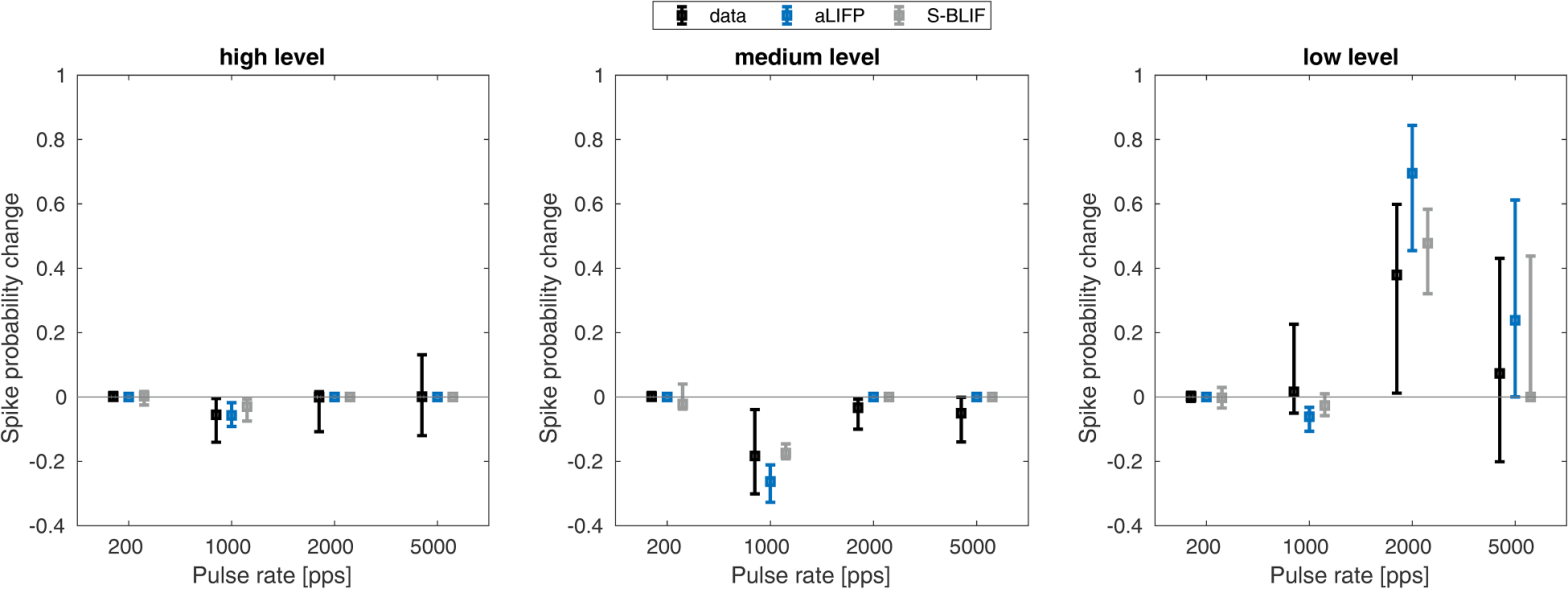
Facilitation and accommodation as calculated from spike trains by Heffer et al. (2010). Negative values indicate accommodation, while positive ones denote facilitation. The predictions from both the S-BLIF and the aLIFP model fit the data nicely, except for 2,000 pps at low levels, where the aLIFP model overestimates the facilitation. aLIFP = adaptive leaky-integrate and firing probability; S-BLIF = sequential biphasic leaky-integrate and fire.

While both models contain facilitation, only the aLIFP model has accommodation incorporated. However, the S-BLIF model has an accommodation-like response to biphasic pulses, due to the leaky integrator, as explained previously. This accommodation-like behavior can be seen when modeling the data by Heffer et al. (2010). They measured the response of 144 guinea pig auditory nerve fibers to pulse trains containing biphasic pulses with a phase duration of 25 µs and an IPG of 8 µs. Then, they obtained the firing probability in the first 2 ms from the data and compared it to the linearly predicted probability from the first pulse. The prediction is done by multiplying the spike probability of the first pulse with the number of pulses presented. If the predicted probability is lower than the measured one, it indicates accommodation, and if it is higher, it indicates facilitation. This was done for three different spike probability ranges, which were taken from the original publication (low: 0.02–0.18, medium: 0.3–0.7, and high: 0.75–0.98). We would like to point out that the approach by Heffer et al. (2010) overestimated the amount of accommodation, as discussed by Takanen and Seeber (2022b). The experimental results, together with the predictions from both models, can be seen in Figure 8. Both models accurately simulate the data, even though the reasons for the decreased spike rate differ. Only the facilitation predicted by the aLIPF model at a low level and 2,000 pps is slightly too high, while the S-BLIF mode can reproduce the data more closely. The difference in the prediction is again due to the fitting of different facilitation data. As the data by Dynes (1996) were obtained from only a single nerve fiber, it is possible that it does not represent the average response to subthreshold stimulation.

**Figure 9.**
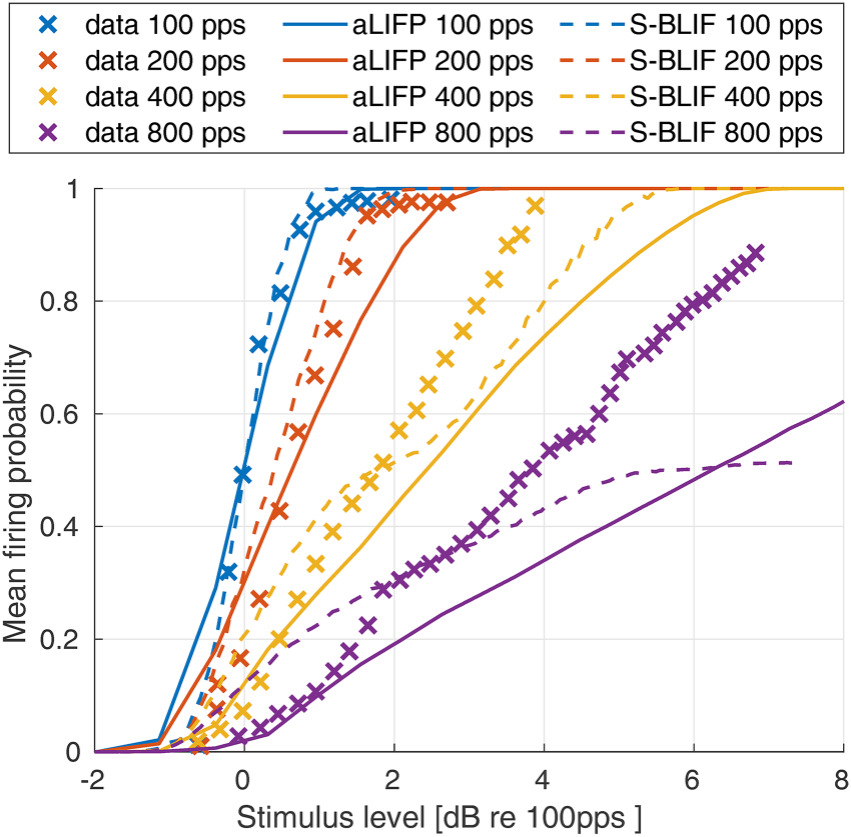
Mean firing probability over level for four 100 µs pulse trains with different pulse rates. The *x*-axes give the amplitude in dB relative to the $I_{50}$ at 100 pps. The data were collected on a single cat nerve fiber by Javel (1990) and simulated using both models. The graphs show the predictions of both the aLIFP model and the S-BLIF model. aLIFP = adaptive leaky-integrate and firing probability; S-BLIF = sequential biphasic leaky-integrate and fire.

Javel (1990) measured the mean spike probability of a single cat auditory nerve fiber for a 100 ms pulse train with four different pulse rates. The biphasic pulses had a phase length of 50 µs and no IPG. The data, together with the predictions from the aLIFP and the S-BLIF models, can be found in Figure 9. Both models capture the general trend of the data, which is a shallower slope of the rate-level function with a rising pulse rate. However, both models underestimate the mean spike probability, and this difference also becomes larger with a higher pulse rate. During the fitting process, we have found that the predictions of this data set are highly dependent on the refractory and adaptation parameters. This indicates the possibility that the single cells used by Zhang et al. (2007) and Javel (1990) have different adaptive properties, and that the aLIFP model could predict the data from Javel (1990) if another set of adaptation parameters were used.

**Figure 10.**
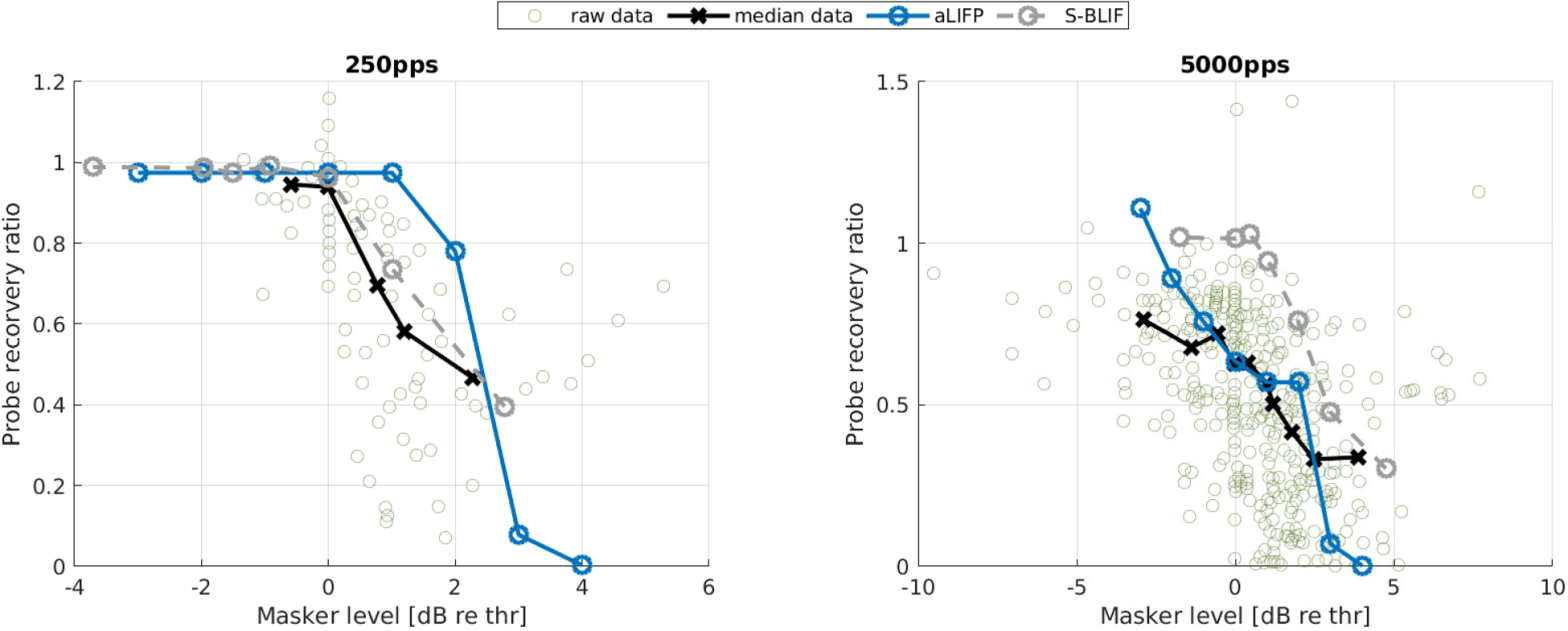
Data collected by Miller et al. (2011), who measured the impact of a low amplitude stimulus on a subsequent probe in 48 cat nerve fibers. The amplitudes were placed around the spike threshold for the masker, defined as the level where firing just started. For the 250 pps stimulus, the S-BLIF mode fits the data more closely, while for the 5,000 pps stimulus, the predictions from the aLIFP model are closer to the data. aLIFP = adaptive leaky-integrate and firing probability; S-BLIF = sequential biphasic leaky-integrate and fire.

Miller et al. (2011) evaluated the effect of a low-amplitude masker on the firing probability of a probe in 48 cat auditory nerve fibers. For this, they defined the probe recovery ratio as the masked total spike probability of the probe divided by the unmasked one. The amplitudes of the masker were placed around the current amplitude, where the nerve fiber starts firing. The masker had a length of 200 ms, with a pulse rate of either 250 pps or 5,000 pps, and the probe had a length of 250 ms and a rate of 100 pps. All pulses were biphasic and had a phase length of 40 µs and an IPG of 30 µs. To simulate the Miller et al. (2011) data, we used a level of 0.9 mA (−2.7 dB re single pulse $I_{50}$ _)_ as the spike threshold for our model. The probe level was set to 1.1 mA (−1.4 dB re single pulse $I_{50}$ _)_. Like in the original publication, the spike threshold is chosen as the stimulus amplitude for which the modeled nerve fiber starts spiking, and the relative distance to the probe level was kept constant. For the S-BLIF model, the same values as in their original publication (Takanen & Seeber, 2022b) were used. The raw data and the median data for a 250 pps and a 5,000 pps masker are shown in Figure 10, together with the predictions from the aLIFP model and S-BLIF model. A higher amplitude leads to higher masking in both cases, which can be attributed to refractory and adaptation effects. Both models reproduce this trend. For the 250 pps stimulus, the S-BLIF model is closer to the data. However, for the 5,000 pps pulse train, the S-BLIF model is not able to reproduce the masking for stimuli below the spike threshold. As our aLIFP model has accommodation incorporated, it reproduces the decrease in the probe recovery ratio to some extent. For higher masker levels, the aLIFP model overestimates the decrease in probe recovery ratio but stays in the range of the raw data points.

The synchrony of the spikes to the stimulus can be expressed using vector strength (Goldberg & Brown, 1969). Usually, this measure is calculated using spike times from multiple runs or recordings. Here, the calculations are done on the sampled spike time distribution $D_{\mathrm{st}}$ instead of the individual spike times, resulting in

(34) $$\mathrm{VS}=\frac{1}{\sum_{i=1}^{N}\;p_{i}}\sqrt{{\left[\overset{N}{\underset{i=1}{\sum }}\;p_{i}\cos\;\left(\frac{2\pi t_{i}}{T}\right)\right]}^{2}+{\left[\overset{N}{\underset{i=1}{\sum }}\;p_{i}\sin\;\left(\frac{2\pi t_{i}}{T}\right)\right]}^{2}}\;\;.$$

The sampled firing probability at the time point $t_{i}$ is $p_{i}$ and *T* expresses the interpulse interval in the stimulus. For the calculation, the sampling interval size of the firing probability was chosen as 1 µs.

The vector strength was predicted and compared to data from Miller et al. (2008), Dynes and Delgutte (1992) and Hartmann and Klinke (1990). The data from Dynes and Delgutte (1992; 120 auditory nerve fibers in 11 cats) and Hartmann and Klinke (1990; 54 auditory nerve fibers in three cats) were measured with sinusoidal stimulation instead of pulse trains. However, as they are in good agreement with the data by Miller et al. (2008), they were included in this work. Miller et al. (2008) measured the neural synchrony in 88 fibers of three cats using biphasic pulse trains. They had a phase duration of 40 µs, however, the length of the IPG was not given. Therefore, we predicted the data for two IPG durations: 0 µs and 30 µs.

**Figure 11.**
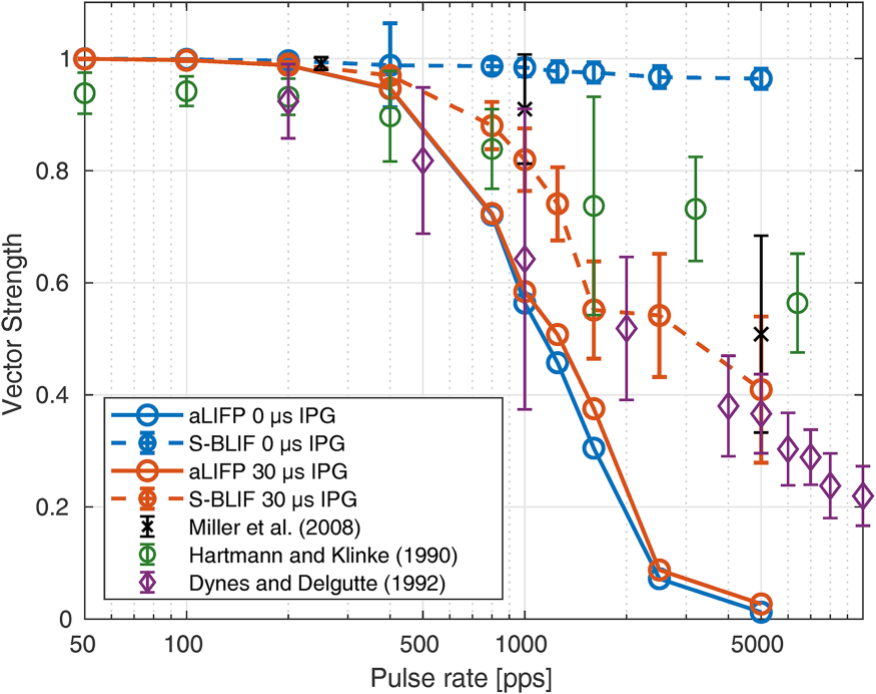
The vector strength from three different publications for different pulse rates measured in cats. As the duration of the IPG was not clear, the predictions were made both for an IPG of 0 µs and 30 µs. For the S-BLIF model, the stimulus amplitude was slightly increased for the 2500 pps and 5000 pps pulse trains. The predictions of the aLIFP model are not affected by the IPG and it fits the data for both pulse shapes up to 1600 pps. The S-BLIF model only fits the data with 30 µs IPG very well but cannot reproduce the data with an IPG of 0 µs. aLIFP = adaptive leaky-integrate and firing probability; S-BLIF = sequential biphasic leaky-integrate and fire; IPG = interphase gap.

The stimulus amplitude for our predictions was chosen to achieve 90% firing probability for a single pulse. For the S-BLIF model, the same amplitudes as in the original publication have been used. As can be seen in the orange lines in Figure 11, the S-BLIF model can reproduce the data for pulses with 30 µs IPG very well, while the aLIFP model struggles for pulse rates above 1,600 pps. This is the case because the jitter becomes too large for small spike probabilities. Our model can predict too large jitter values because we defined the jitter over the distance between membrane potential and the mean threshold, instead of the spike probability as in the S-BLIF model. The model predictions of the vector strength indicate that our definition of the jitter might be too simplified, and the true jitter definition is somewhere between ours and the one by Horne et al. (2016).

For the case without an IPG, the predictions of the aLIFP model stay similar, however, the vector strength predicted by the S-BLIF model is too high and always close to one, as can be seen in the blue lines in Figure 11. The reason for this difference lies in the interaction between jitter and spike cancellation. In the S-BLIF model, the spike cancelation mechanism leads to a very low jitter for pulses with short IPG, which then causes a high vector strength. This is illustrated in more detail in the Appendix.

In the last tested data set, the impact of amplitude modulation on spike rate and vector strength was examined. For this, Hu et al. (2010) presented 5,000 pps pulse trains to 72 cat auditory nerve fibers. The biphasic pulses had a phase duration of 40 µs and no IPG. Both the spike rate and vector strength for unmodulated pulse trains and for modulated ones were obtained. For the modulated pulse trains, the vector strength was obtained with respect to the modulation frequency, while for the unmodulated pulse trains, it was obtained relative to the stimulation frequency. The modulated stimulus was obtained by modulating an unmodulated pulse train p(t) via

(35) $$(1+m\cdot \sin\;(2\pi \cdot f_{m}\cdot t))\cdot p(t)\;.$$

**Figure 12.**
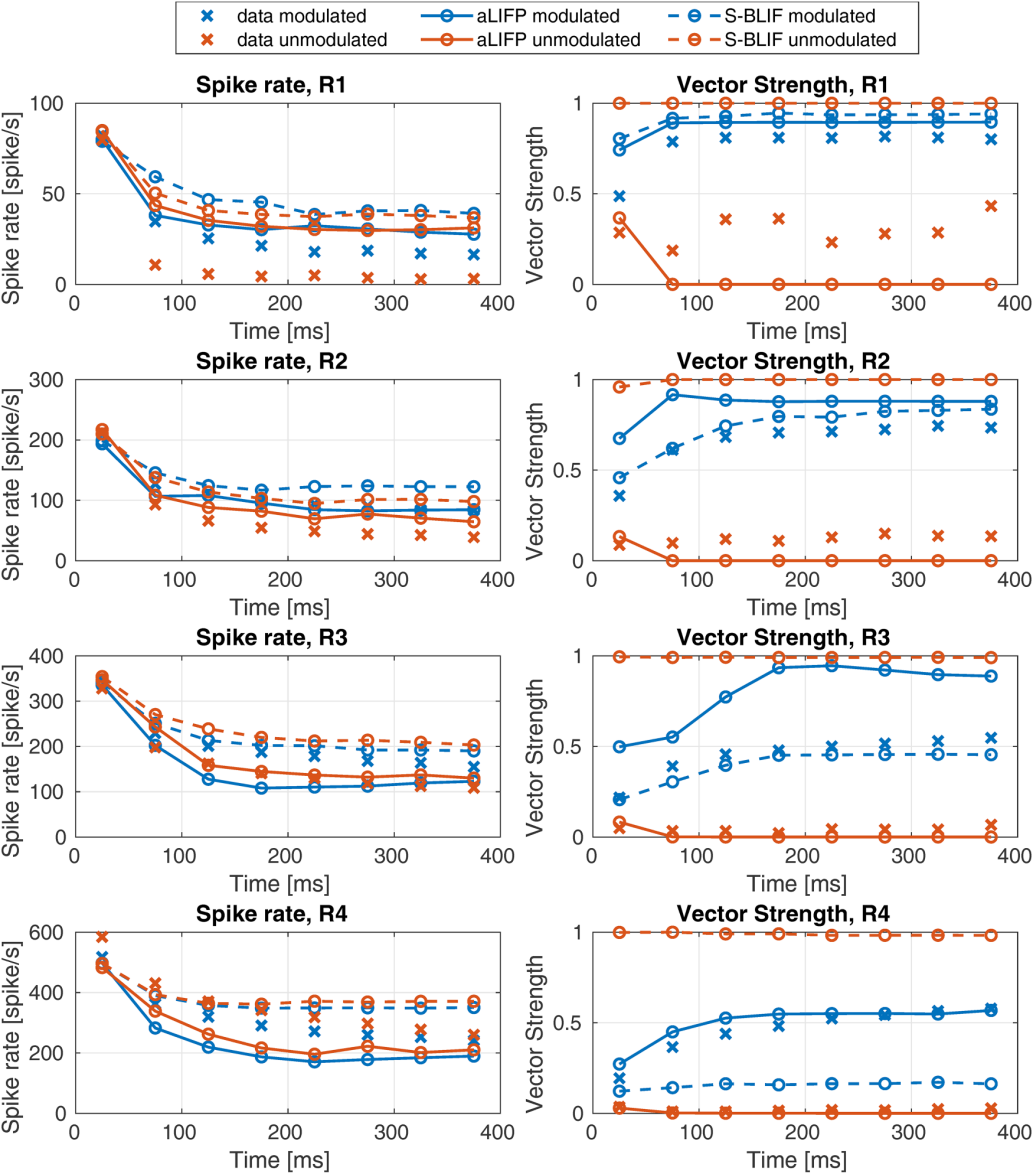
The spike rate and the vector strength of 100 Hz sinusoidally amplitude modulated and unmodulated pulse trains with a rate of 5,000 pps were calculated. The predictions of both the aLIFP model and the S-BLIF model were compared to data of 72 cat nerve fibers from Hu et al. (2010). Both models can predict parts of the dataset, which are not always the same. aLIFP = adaptive leaky-integrate and firing probability; S-BLIF = sequential biphasic leaky-integrate and fire.

The modulation frequency $f_{m}$ was 100 Hz and the modulation depth *m* was 0.1. The results of the fibers were grouped by Hu et al. (2010) depending on their spike rate in the first 50 ms. The group R1 contains fibers with 5–150 spikes/s, R2 with 150–270 spikes/s, R3 with 270–400 spikes/s, and R4 with 400–972 spikes/s. The stimulation amplitudes for the models were chosen such that they produce the mean spike values in the first time bin for the four groups. As the grouping was done independently for the modulated and unmodulated stimuli, the model amplitudes were also chosen separately for both. It can be seen in Figure 12 that both models can reproduce the decay in spike rate for the rate groups R2–R4 groups in the modulated case, but not for the R1 group, where the aLIFP model overestimates the spike rate after the onset. The S-BLIF model also overestimates the spike rate slightly but can reproduce the trend. The trend of decreasing vector strength for higher spike rates in the modulated cases can be reproduced by both models, with the S-BLIF model fitting groups R2, and R3 better and the aLIFP model group R4. Both models perform similarly for the first group.

In the unmodulated case, shown in Figure 12 in orange, both models overestimate the spike rate for group R1 hugely and slightly for group R2. For the spike groups R3 and R4, they can predict the decay in spike rate over time. The vector strength is predicted by the aLIFP model as a very low value, which is consistent for R2, R3, and R4, but not R1. The S-BLIF model, on the other hand, predicts a vector strength of 1, which is much too high. This is related to the influence of the IPG on the vector strength, which is discussed in the Appendix. In summary, both models predict the main trends for the modulated stimulus but have some problems predicting the vector strength of the unmodulated stimulus.

## Discussion

### Summary of Main Findings

The proposed aLIFP model is a phenomenological model of the electrically stimulated auditory nerve fiber. It directly predicts the firing rate over time in response to biphasic pulses instead of providing stochastic spike times. As it was our aim to provide the instantaneous firing probability as an alternative model output, not to improve the overall predictions, our model is heavily based on the S-BLIF model (Takanen & Seeber, 2022b). We have shown that for biphasic pulses with a cathodic leading phase, the performance of both models is similar.

To describe the stochasticity of the cell response, Takanen and Seeber (2022b) use a noisy threshold drawn from a time-varying Gaussian distribution and draw the latency between the threshold crossing and the spike time from a Gaussian distribution. Our model describes the threshold and the latency using the same distributions, but instead of drawing from these distributions, all calculations are directly performed on them. This allows us to predict the spike probability over time, described by one or more Gaussian distributions, in response to each pulse. Providing the spike probability over time has the advantage of a deterministic model output, which allows to observe the influence of small parameter or input changes onto the output. One could argue that the comparison with real-world spike time data is reduced, as the output is provided in a different modality. However, statistical tools, such as maximum likelihood, exist to evaluate the probability that samples are provided by a distribution. When comparing spiking model output with spiking data, one must resort to heuristic metrics describing the distance between two spike trains (e.g., Victor & Purpura, 1997) and also consider the stochasticity of the response or calculate summary statistics on multiple repetitions. The latter is also facilitated by our nonspiking model, as it directly provides the summary statistics.

Additionally, the nonspiking approach decreases the runtime of the simulation if the instantaneous firing probability or spike rate is the value of interest, as it only needs to be run once to obtain all required average rates and other response measures. Table 2 shows a comparison of the runtimes of the S-BLIF and the aLIFP model for a single pulse and a stimulus with a length of 0.1 s and 100 pulses at three amplitudes. Comparing the runtimes, for a single pulse and for a high amplitude 100-pulse stimulus, the aLIFP model is faster, while for a 100-pulse stimulus with a low or medium amplitude, the S-BLIF model is faster. However, to obtain an estimate of the spike probability, the S-BLIF model needs to be run multiple times, and the aLIFP model is faster for all conditions than running the S-BLIF model 100 times, assuming that this is a reasonable sample to estimate the spike probability. For both models, the runtime is dependent on the amplitude of the stimuli. For the aLIFP model, the runtime increases with the number of paths. Therefore, the number of paths increases with the number of presentations that lead to a spike with a probability larger than 0 and smaller than 1. To avoid an exponential increase of the runtime, the number of paths is limited. For the current implementation, the limit is 20, corresponding to an average probability of 0.05 of a single path. In the S-BLIF model, the runtime depends on the number of spikes. Therefore, for a very high amplitude, the runtime of the S-BLIF model is much higher compared to the aLIFP model. The latter has the highest runtime for intermediate amplitudes.

**Table 2.** Runtime of Both Models for a Single Pulse and a 0.1 s Stimulus With 100 Pulses at Three Different Amplitudes.

Model	Single pulse (ms)	100 pulses, amplitude $\ll I_{50}$ (s)	100 pulses, amplitude $\approx I_{50}$ (s)	100 pulses, amplitude $\gg I_{50}$ (s)
aLIFP	0.4	0.3	4.1	0.3
S-BLIF	0.55	0.01	1.6	2.5

*Note*. aLIFP = leaky-integrate and firing probability; S-BLIF = sequential biphasic leaky-integrate and fire.

To test our model, it was fitted and validated on single cell recordings of electrically stimulated auditory nerve fibers from cats and guinea pigs stimulated with biphasic pulses. As our model can only be excited by a cathodic (negative) phase, we have always used cathodic-leading stimuli, independent of the ones used to collect the data. Within these limitations, we were able to fit our parameters to the literature data and could demonstrate the generalizability of our model to other data.

### Relationship to Existing Models

Using the spike probability in nerve fiber models is not a novel idea. Goldwyn et al. (2012) calculated the instantaneous spike probability in their point process model by applying a cascade of filters to the model input. This approach differs completely from the one used in our model and results in a complex distribution for the instantaneous spike probability, which has the maximum at the beginning and then decays exponentially. In contrast to this, our model describes the instantaneous spike probability using one or more Gaussian distributions. When looking at the neuronal recordings by Javel and Shepherd (2000), the distribution of the spike times is neither exponentially decaying nor a Gaussian distribution. Instead, Javel and Shepherd (2000) described the distribution of a single cell using a sum of multiple, slightly skewed Gaussian distributions, with the maximum peak at the beginning. Therefore, both the description with an exponentially decaying distribution and with a sum of multiple Gaussian distributions are reasonable approximations of the spike distribution. To increase the physiological plausibility of our model, one can start with multiple components in the threshold, which then leads to multiple Gaussian distributions in the output, even if the model is stimulated with a single pulse.

In the model by Goldwyn et al. (2012), the instantaneous spike probability in response to a pulse is not used as the output of the model or to compute the pulse interaction phenomena. Instead, a discrete spike time is drawn from the instantaneous spike probability. An extension of this model into a completely nonspiking model is conceivable using our approach of multiple threshold paths. However, the more complex distribution of the instantaneous spike probability would complicate the mathematical description.

Both in the nonspiking model by Xu and Collins (2007) and by Campbell et al. (2012), an integrated Gaussian function is used to relate the amplitude of the input stimulus directly to the spike probability. This is similar to the calculation of the spike probability in the aLIFP model, with the difference that we use the cumulative Gaussian function to create a relationship between the passive membrane potential and the spike probability. Our approach has the advantage of directly describing the influence of the phase length on the spike probability, whereas Xu and Collins (2007) used a separate mechanism and Campbell et al. (2012) did not describe the influence of the phase length.

While our model obtains the spike time distribution in addition to the spike probability neither Xu and Collins (2007) nor Campbell et al. (2012) computed this distribution and only used the total spike probability. This approach is entirely valid and saves computational time if one is interested in the spike probability or rate over a larger time scale and not in the detailed statistics in response to each stimulation pulse.

Our model differs from existing nonspiking models also in the amount of detail of the spike response included. Our model's level of detail is comparable to existing state-of-the-art phenomenological spiking models. For example, neither Xu and Collins (2007) nor Campbell et al. (2012) considered the exact spike timing, and therefore, the latency and the jitter of the response are not included. The point process model by Goldwyn et al. (2012) described the jitter using another filter. On the other hand, both the spiking models by Joshi et al. (2017) and Takanen and Seeber (2022b) considered spike latency and jitter, where Joshi et al. (2017) focused on the difference in latency and jitter between anodic and cathodic stimulation, while Takanen and Seeber (2022b) accurately described the change due to different stimulation amplitudes. As our nonspiking model is based on the latter, we describe the latency and jitter in a similar way.

Data by Shepherd and Javel (1999) have shown that the spike probability of a biphasic pulse is reduced compared to a monophasic pulse. This phenomenon is included in the model by Joshi et al. (2017) inherently, as their model is based on an exponential leaky-integrate and fire model. The linear leaky-integrate and fire model by Takanen and Seeber (2022b) required a separate mechanism for this reduced probability, which they have called the “spike cancellation.” As the aLIFP model is also based on a linear leaky integrator, we have adapted the mechanism by Takanen and Seeber (2022b) for our nonspiking version. Unfortunately, this led to the constraint of biphasic pulses, which is not necessary in the model by Takanen and Seeber (2022b) or Joshi et al. (2017). Studies using nonrectangular pulses (e.g., Ballestero et al., 2015) or triphasic pulses (e.g., Herrmann et al., 2023) can therefore not be simulated with our model. To remove the constraint of biphasic pulses, a more general way of estimating the “spike cancellation” needs to be found. This could also include a more realistic description of the spike prevention by a second phase with the opposite sign, by taking the time dependencies into account.

In nature, both cathodic and anodic pulses can excite a neuron, with different amplitude levels required (Shepherd & Javel, 1999). The model by Takanen and Seeber (2022b) considered stimulation with both polarities using two thresholds, which can be parameterized to different values. However, in the published parameters, both thresholds have been set to the same value. Joshi et al. (2017) considered stimulation with two different polarities in detail. For this, they combine two exponential leaky-integrate and fire models, one for each polarity and fit these in detail with data from the literature. This also facilitates the use of different latency and jitter values for the two polarities. In our model, we have refrained from including this phenomenon, as our focus was on creating a nonspiking model. An extension like the one in the model by Takanen and Seeber (2022b) by using two thresholds could also be incorporated in the aLIFP model.

Four spike interaction phenomena are included in our model: refractory period and adaptation after a spike occurred, and facilitation and accommodation if no spike was evoked. For the refractory period and the adaptation, the same functions as in the model by Takanen and Seeber (2022b) were used. As their model includes facilitation but not accommodation, we have used a new function to model both sub-threshold phenomena with a single function. The model by Joshi et al. (2017) used adaptive currents, which feed back into their description of the membrane potential, to describe all four pulse interaction phenomena instead of changing the threshold potential.

Our model is heavily based on the S-BLIF model by Takanen and Seeber (2022b), and we have therefore included a comparison between both models in the Model Fitting and Model Validation sections. As for some parameters multiple data sets exist, a selection had to be made and the most straightforward choice for our model differs from the one made by Takanen and Seeber (2022b). Unsurprisingly, the data are then resembled more closely by the aLIFP model than by the S-BLIF model. Conversely, some of the data sets that we did not use for fitting have been used to fit the S-BLIF model and are therefore predicted better by the S-BLIF model. For the other validation data sets, the two models often resulted in slightly different predictions, with one model predicting one dataset better and the other model another data set. The overall comparable ability of the aLIFP model and the S-BLIF model is consistent with our aim to create a nonspiking statistical model based on the S-BLIF model, not to improve the prediction accuracy of the S-BLIF model.

### Applications

The deterministic nature of the model and the consequently reduced runtime facilitate its usage in many research areas. For example, the influence of parameter or input changes, however small, can be seen in the model's output. This can be used to investigate the influence of individual parameters on the spiking pattern without the interference of the stochastic output. Furthermore, the easy observation of the influence of parameter changes on the output allows for the fitting of nerve fiber parameters directly onto peristimulus time histograms using a maximum likelihood approach. Additionally, the comparison of coding strategies can be simplified, as the complete spike distribution is provided with a single run and the difference in the output is not obscured by the stochasticity of a single draw from the distribution.

The fast runtime of the aLIFP model facilitates its incorporation into larger models. For example, our model can be combined with a simulation of the human cochlea, such as the one by Nogueira and Ashida (2018), or with the modular framework published by Hu et al. (2023). For the same reason, the incorporation of the model into algorithms, such as coding strategies, is simplified.

The aLIFP model is not limited to auditory nerve fibers or cochlear implant stimulation. The parameters can be fit to any electrically stimulated neuron. The idea of using a Gaussian mixture to describe the phenomenological threshold can also be applied to simulate neural responses to nonelectric stimulation.

## Summary

In this work, we presented an adaptive leaky-integrate and firing probability (aLIFP) model of the electrically stimulated auditory nerve fiber. It is deterministic while still describing the stochasticity of the spike generation process. Therefore, it only needs to be run once to obtain the complete spiking probability over time in response to biphasic pulses, from which the spike rate can easily be calculated. The deterministic nature of the aLIFP model allows researchers to directly observe changes in the output due to small changes in the parameters or input. In combination with the fast runtime, this makes the model predestined for combination with other models or inclusion in algorithms. The aLIFP model was fitted to all data sets using a single set of parameters and can predict the validation data sets with these or at least reproduce the trend in the data.

The aLIFP model is freely available at https://zenodo.org/doi/10.5281/zenodo.11198029 and is going to be part of the next release of the auditory modeling toolbox (Majdak et al., 2022).

## Figures and Tables

**Figure A1.**
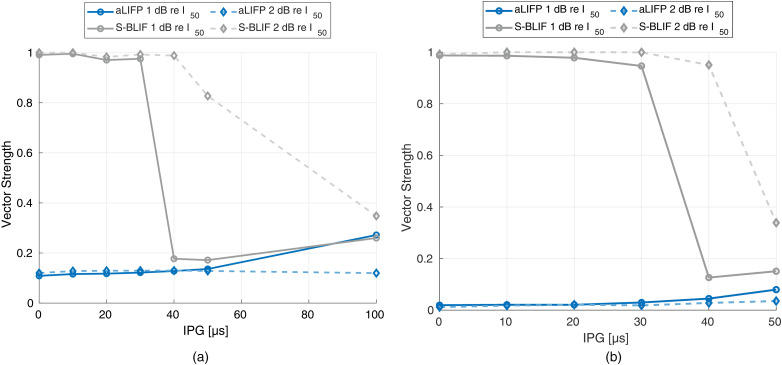
The vector strength obtained from the predictions of both models for different IPG durations and two amplitudes: 1 dB and 2 dB relative to single pulse $I_{50}$ . In (a) the predictions for a 2,500 pps stimulus are shown and in (b) for a 5000 pps stimulus, both with a duration of 0.3 s. For the 5,000 pps stimulus the calculations could only reasonably be performed up to 50 µs IPG, because for higher IPG values the gap between the pulses would have been shorter than the IPG.

## Appendix

The influence of the IPG on the vector strength for both the aLIFP and the S-BLIF model is illustrated in Figure A1, where the vector strength for both models at two amplitudes is plotted over different IPG values. As stimuli, 0.3 s pulse trains with 2,500 pps (a) or 5,000 pps (b) have been used. The pulses had a phase duration of 40 µs For the 2500 pps case in Figure A1a, the vector strength of the S-BLIF predictions is near 1 for IPG durations below 20 µs and decays fast afterwards. For the aLIFP model, the vector strength stays similar across the IPG. As already stated, the influence of the IPG on the vector strength in the S-BLIF model is because the spike cancellation is not considered in the calculation of the jitter. Therefore, for a short IPG, a pulse which evokes spikes with low probability still results in a small amount of jitter and thus a high vector strength. In the aLIFP model, spike cancellation is considered in the calculation of the jitter, and therefore, the IPG only has a small effect on the vector strength. Unfortunately, no physiological data were found that describes the vector strength, or a similar measure, for different IPG durations and levels. Therefore, we cannot compare the predictions to the data and decide which mechanism leads to better predictions.

The 5,000 pps predictions in Figure A1b show a similar trend for both models. For this pulse rate, the predictions were only performed up to 50 µs IPG because the interpulse time is only 200 µs and the silent period only is 120 µs. Already at an IPG of 60 µs, the duration between the pulses is as short as the IPG. Effectively, this leads to pulses with a cathodic leading phase and swapped IPG and interpulse duration.
